# Special Issue on “Proteostasis and Autophagy”

**DOI:** 10.3390/cells8070642

**Published:** 2019-06-26

**Authors:** Andreas Kern, Christian Behl

**Affiliations:** Institute of Pathobiochemistry, University Medical Center of the Johannes Gutenberg University, Duesbergweg 6, 55128 Mainz, Germany

Autophagy is a highly conserved eukaryotic pathway responsible for the lysosomal degradation (and subsequent recycling) of cellular components such as proteins, protein aggregates, and a growing number of organelles or cellular compartments [1]. Since autophagy was first described in the 1960s, the identification of autophagy-related genes, beginning in the 1990s, has boosted the field, which is rapidly growing and is elucidating an intriguing mechanistic complexity as well as a tremendous range of cargo substrates.

Besides autophagy, the ubiquitin proteasome system (UPS) is a further potent proteolytic system within the cell [2]. Both degradation processes are essential for cellular protein homeostasis (proteostasis) and thus are fundamental for cellular vitality. Imbalances in proteostasis are connected to aging and multiple (age-associated) disorders.

The articles in this Special Issue (original papers and reviews) focus on the importance of distinct autophagy factors, regulators and modifiers or of the autophagy network as a whole for the efficient degradation of proteins and organelles; e.g., mitochondria. Some contributions aim at the crosstalk of autophagy and the UPS, depicting the close functional association of both systems. As a common feature, the articles combine the dysfunction of autophagy with disturbances in proteostasis and link it to aging and several disorders, including neurodegenerative disease, cancer, inflammation, infection, and cardiac disease (Figure 1).

- Three reviews focus on the interplay of UPS and autophagy. Xiong and colleagues address the role of ATG16 in autophagy and the UPS and possible links to inflammatory disease and infection [3], while Zientar-Rytter and Subramani summarize the current knowledge on the ubiquitin recognition code and ubiquitin binding of proteasomal and autophagic receptors [4]. Wiegering and colleagues in their review aim to resolve the mechanism behind the crosstalk of the two degenerative pathways and on the particular role of primary cilia structures in this interplay [5].
- The review of Dong and Cui displays the role of the autophagy–lysosomal pathways in the modulation of proteostasis in tumorigenesis and cancer development [6]. More specifically, Zada and colleagues present original data on the control of epithelial-to-mesenchymal transition and cancer metastasis by the autophagy-dependent degradation of the nuclear protein SNAI1 [7].
- By focusing on the role of autophagy in cardiac disease, the review presented by Li and colleagues deals with the interplay between autophagy and proteostasis in the healthy heart and the consequences of an unbalanced proteostasis in cardiomyocytes [8].
- In a more general overview, the review by Chun and Kim summarizes findings on the significance of autophagy as an essential degradation program for cellular homeostasis and life in general, also addressing recent advances in post-translational modifications of autophagic proteins and autophagy as therapeutic target [9].
- Original data by Fischer and colleagues show the functional characterization of the ubiquitin-like core autophagy protein ATG12 in the social amoebae *Dictyostelium discoideum*, presenting evidence on autophagy-independent functions of ATG12 and ATG16 in addition to their role in canonical autophagy [10]. The original paper by Takacs et al. deals with the two homologs of the autophagic WIPI proteins ATG-18 and EPG-6 in the nematode *C. elegans* and shows that both proteins are required for autophagy and also differentially contribute to the control of the lifespan of this worm [11].
- Finally, a collection of papers presents original work and reviews on the impact of autophagy on neuronal models and neurodegeneration. Zveronik presents data on the human protease inhibitor Stefin B, which, upon overexpression, forms protein aggregates which induce autophagy [12]. Liang reviews emerging concepts and functions of autophagy as a regulator of synaptic components, plasticity and memory formation [13]. Reddy and Oliver in their review highlight recent developments of the Alzheimer disease-associated amyloid beta and tau protein and their role in modulating autophagy and mitophagy, the autophagic clearance process of defective mitochondria [14]. This presentation is complemented by the review work of Oikawa and Walter by focusing on the function of Alzheimer-related presenilins and gamma-secretase in the protein homeostasis of membranes [15]. By extending the role of autophagy also to other neuropsychiatric disorders, Rein in his review discusses the hypothesis that autophagy contributes to the effect of pharmacological antidepressants beyond the treatment of depression [16]. Finally, the original data by Christ and colleagues show for the first time that the activation of the sigma-1 receptor with selective experimental ligands directly induces autophagy in human cells and in *C.elegans* [17].

We hope that the Special Issue will attract readers from a wide range of specializations, will fuel discussion on this hot research topic, and will emphasize the understanding of the crucial role of autophagy for the maintenance of cellular proteostasis and, if disturbed, its contribution to the pathogenesis of disease.

## Figures and Tables

**Figure 1:**
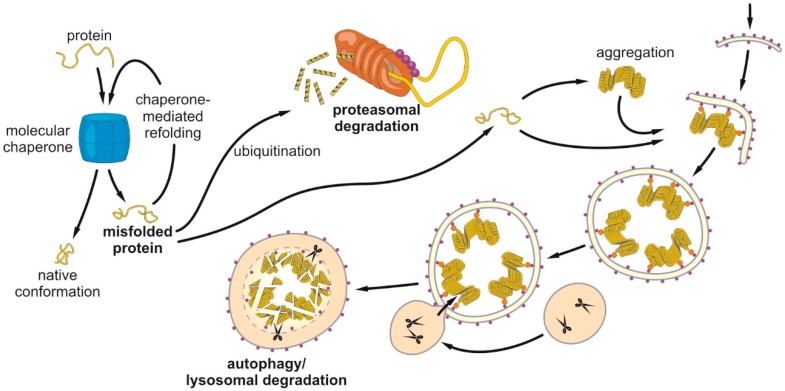
The two key cellular protein degradation pathways to ensure protein homeostasis: proteasomal degradation and the autophagic–lysosomal pathway. Protein folding is controlled by chaperones to ensure a correct three-dimensional protein structure. Misfolded proteins can be transferred to two key principal degradation pathways. While proteasomal degradation represents mostly the routine degradation of dysfunctional proteins including regular protein turnover, the autophagosomal pathways clear cargo such as protein oligomers and aggregates (disease-proteins), whole organelles (mitochondria), intruders (upon bacterial or viral infection) but also membrane fragments (endoplasmatic reticulum).
